# A model of collective behavior based purely on vision

**DOI:** 10.1126/sciadv.aay0792

**Published:** 2020-02-05

**Authors:** Renaud Bastien, Pawel Romanczuk

**Affiliations:** 1 Department of Collective Behavior, Max Planck Institute for Ornithology, Konstanz, Germany.; 2 Department of Biology, University of Konstanz, Konstanz, Germany.; 3 Department of Biology, Institute for Theoretical Biology, Humboldt Universität zu Berlin, Berlin, Germany.; 4 Bernstein Center for Computational Neuroscience, Berlin, Germany.

## Abstract

Classical models of collective behavior often take a “bird’s-eye perspective,” assuming that individuals have access to social information that is not directly available (e.g., the behavior of individuals outside of their field of view). Despite the explanatory success of those models, it is now thought that a better understanding needs to incorporate the perception of the individual, i.e., how internal and external information are acquired and processed. In particular, vision has appeared to be a central feature to gather external information and influence the collective organization of the group. Here, we show that a vision-based model of collective behavior is sufficient to generate organized collective behavior in the absence of spatial representation and collision. Our work suggests a different approach for the development of purely vision-based autonomous swarm robotic systems and formulates a mathematical framework for exploration of perception-based interactions and how they differ from physical ones.

## Teaser

From minimal visual information, organized collective behavior can emerge without spatial representation or collisions.

## INTRODUCTION

Models of collective behavior often rely on phenomenological interactions of individuals with neighbors [e.g., see (*1*–*8*)]. However and contrary to physical interaction, these social interactions do not have a direct physical reality, such as gravity or electromagnetism. The behavior of individuals is influenced by their representation of the environment, acquired through sensory information. Current models often suggest that individuals are responding to the state of movement of their neighbors, their (relative) positions and velocities, which are not explicitly encoded in the sensory stream. Thus, these phenomenological interactions implicitly assume internal processing of the sensory input to extract the relevant state variables. On the other hand, neuroscience has made tremendous progress in understanding various aspects of the relation of sensory signals and movement response, yet connections to large-scale collective behavior are lacking. Although evidence has been found for neural representation of social cues in the case of mice (*9*) and bats (*10*), details and role of these internal representations remain unclear, particularly in the context of coordination of movement. Collective behavior crucially depends on the sensory information available to individuals; thus, ignoring perception by relying on ad hoc rules strongly limits our understanding of the underlying complexity of the problem. Besides, it obstructs the interdisciplinary exchange between biology, neuroscience, engineering, and physics.

Recently, the visual projection field has appeared as a central feature of collective movements in fish (*11*–*14*), birds (*15*), humans (*16*), or artificial systems (*17*, *18*). Because of the geometrical nature of vision, i.e., the projection of the environment, vision appears as a good starting point to explore the relationship between sensory information and emergent collective behaviors. Some models have attempted to relate vision and movement (*4*, *15*, *17*, *19*). However, they use vision as a motivation to refine established social interaction models or rely on additional interactions based on information not explicitly represented in visual input such as distance or heading direction of neighboring individuals. Furthermore, most of the above models consider only part of the interaction by assuming constant speed of individuals and focusing solely on their turning response. A more general modeling approach is required to investigate the role of adaptive speed in vision-mediated movement coordination.

Here, we propose a radically different approach by introducing a general mathematical framework for purely vision-based collective behavior. We use a bottom-up approach using fundamental symmetries of the problem to explore what types of collective behavior may be obtained with as minimal as possible requirements.

## MATERIALS AND METHODS

**Fig. 1:**
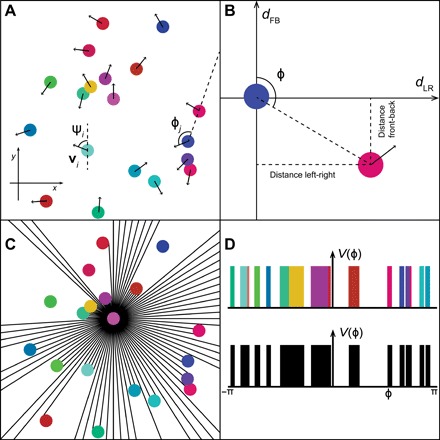
Geometry of the system. (**A**) A set of disks with diameter BL (body length) is considered. Each disk is propelled in the direction ψ*_i_* with a velocity *v_i_*(*t*). (**B**) A co-moving referential can be defined following the movement of the disk *j*. This referential is centered on the position of the disk, (*x_j_*,*y_j_*), and oriented so that the vertical axis is aligned with the direction ψ*_j_*. The position of other objects can be recovered through their left-right (*d*_LR_) and front-back (*d*_FB_) distances relative to the disk *j*. ϕ represents the swiping angle. (**C**) Representation of the visible field of the pink disk through ray casting. The position of the eye is considered to be at the center of the disk with a fully circular point of view, i.e., no blind angles. (**D**) The projection of the visual field in two dimensions (2D) is given by a 1D function. On top, objects can be represented by their colors. However, on the bottom part, a binary visual field is given. It is not possible to distinguish individuals.

Formally, we can write the movement response of an agent to the visual projection field *V* in three spatial dimensions as the following evolution equation for its velocity vector **v***_i_* (see Fig. 1 for the geometry of the problem)

$$\partial_{t}v_{i}(t)=F_{\text{ind}}(v_{i})+F_{\text{vis}}[V_{i}(\phi_{i},\theta_{i},t)]$$ (1)

The first term accounts for the self-propelled movement of an individual. Here, we used a simple linear propulsion function: **F**_ind_ = γ[*v*_0_ − *v_i_*(*t*)]**vˆ***_i_*(*t*), with *v*_0_ being the preferred speed of an individual, γ being the speed relaxation rate, and **v**ˆ*_i_* being the heading direction vector of the focal individual with |**v**ˆ*_i_*| = 1. The second term accounts for the movement response to the visual sensory input given by the visual projection field *V_i_*(ϕ*_i_,*θ*_i_,t*) experienced by the individual *i*. ϕ*_i_* and θ*_i_* are the spherical components relative to the individual *i*, and **F**_vis_ is an arbitrary transformation of the visual field. This function does not have an explicit dependence on the other individual properties.

The physical, visual input corresponds to a spatiotemporal intensity and frequency distribution of the incoming light. In our framework, we considered *V* to be an abstract, arbitrary representation of the visual input. In particular, *V* can implicitly account for relevant sensory (pre-)processing, e.g., it can represent colors or brightness of the visual scene. Furthermore, *V* can also account for higher-order processing of visual stimuli such as object identification and classification. Equation 1 describes the projection of the full information encoded in the visual field onto the low-dimensional movement response and must hold for any particular choice of visual field.

To simplify the description, we limited our analysis first to the two-dimensional (2D) case. Without any loss of generality, **F**_vis_ can be written as

$$F_{\text{vis}}[V]=\int_{-\pi }^{\pi }d\phi_{i}G[V(\phi_{i},t)]h(\phi_{i})$$ (2)

The functional *G*[*V*] encodes what information from the visual input influences the movement response and how. An arbitrary *G* can be expanded as a series of derivative in space and time and power series of the visual field. This accounts for any function of the visual projection field, e.g., specific functions of the visual cortex such as detection of edges in all directions or optical flow. The function *h*(ϕ*_i_*): ℝ → ℝ^2^, on the other hand, encodes the fundamental properties of the perception-motor system (“the observer”) independent of the specific visual input, e.g., symmetries of the movement response, or spatial dependence of perception (e.g., blind angle). Experimental data in fish have shown that the variation of orientation depends on the left-right position of the other individual, while variations of speed depend on the front-back position. The components of *h* are therefore expanded as a Fourier series in ϕ.

Up to this point, no approximation has been made; the model is as general as possible regarding response to an arbitrary visual field. To develop a systematic understanding of how collective behavior can arise from the visual field, we proposed a minimal model of vision-based interactions. First, we assumed that individuals respond to an instantaneous, binary visual field, i.e., the visual projection field *V* (ϕ*, t*) only accounts for the presence or absence of objects and no other properties. Second, we considered an expansion of an arbitrary functional *G* in terms of the lowest order of retinal space and time derivatives in *V*. The velocity vector of an agent in 2D is determined by the velocity with respect to the heading direction *v_i_*(*t*) and the polar angle determining the heading vector ψ*_i_*(*t*). The simplest equations of movements, satisfying the fundamental symmetries from (*20*), read

$$\begin{array}{c} \partial_{t}v_{i}(t)=\gamma (v_{0}-v_{i}(t))+\\ \int_{-\pi }^{\pi }d\phi_{i}\text{cos}\phi_{i}\alpha_{0}(-V_{i}(\phi_{i},t)+\alpha_{1}{\left(\partial_{\phi i}V_{i}(\phi_{i},t)\right)}^{2}+\alpha_{2}\partial_{t}V_{i}(\phi_{i},t)) \end{array}$$ (3)

$$\partial_{t}\psi_{i}(t)=\int_{-\pi }^{\pi }d\phi_{i}\text{sin}\phi_{i}\beta_{0}(-V_{i}(\phi_{i},t)+\beta_{1}{\left(\partial_{\phi i}V_{i}(\phi_{i},t)\right)}^{2}+\beta_{2}\partial_{t}V_{i}(\phi_{i},t))$$ (4)

The first terms in the brackets in the integral describe the movement response to the perceived angular area (subtended angle) of the objects in the visual projection; the second ones describe the response to edges, while the third ones account for dynamical changes such as translation or loom. The coefficients α*_m_* and β*_n_* are arbitrary constants obtained from the expansion of *G*. In the following, we showed that coordinated collective movement can also emerge without considering temporal derivatives, i.e., by setting α_2_ = β_2_ = 0. In the following, our analysis is restricted to a simple case where only a binary projection of the visual field is considered (Fig. 1, C and D).

## RESULTS

**Fig. 2:**
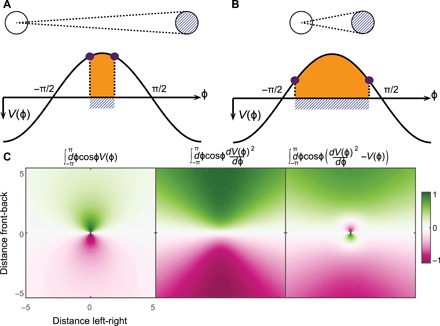
Effects of the terms of Eq. 3 on a focal observer according to the relative position of another disk. (**A** and **B**) The white disk is looking straight at the blue disk with an eye positioned in the center. When the object is far (A), the subtended angle of the object on the projection of the visual field, *V* (ϕ), is smaller than when the object is close (B). When integrating with a cosine function, the subtended angle of the object (orange) results in a larger integration for a closer object, while the edges (purple) sum larger elements of the cosine when the object is far. (**C**) For different relative positions between both disks (Fig. 1B), the subtended angle of the object produces a short-range interaction, while the edges create a long-range interaction. The difference of those two terms can create a short-range repulsion (with the subtended angle of the object)/long-range attraction (with the edges of the object).

The first terms associated with the angular area of objects in the visual projection creates a short-range interaction that decreases as the object gets further (Fig. 2 and fig. S4). On the contrary, the second terms with the first derivative with respect to the visual field coordinate yield long-range interaction due to the nonlinearity of the sine/cosine function (Fig. 2 and fig. S4). Thus, these lowest-order terms, neglecting temporal derivative, are sufficient to generate short-range repulsion and a long-ranged attraction: The individual is repelled by the subtended angle of the object on its visual field while getting attracted by the edges. On the basis of the choice of corresponding interaction parameters, we can define an equilibrium distance, where attraction and repulsion balance (see the Supplementary Materials for details). This equilibrium now introduces a characteristic metric length scale into the system despite the lack of any representation of space at the level of individual agents.

The front-back equilibrium distance is $L_{\text{eq}}^{\left(\text{fb}\right)}=\text{BL}/2\alpha_{1}$, whereas the left-right equilibrium distance is $L_{\text{eq}}^{\left(\text{lr}\right)}=\text{BL}/2\beta_{1}$, with BL (body length) being the diameter of individual agents. Here, we will focus on the case α_1_ = β_1_, i.e., where the attractive terms associated with the edges are equal for turning and acceleration, resulting in spatially isotropic equilibrium distance $L_{\text{eq}}=L_{\text{eq}}^{\left(\text{fb}\right)}=L_{\text{eq}}^{\left(\text{lr}\right)}$ in 2D (see the Supplementary Materials for details).

**Fig. 3:**
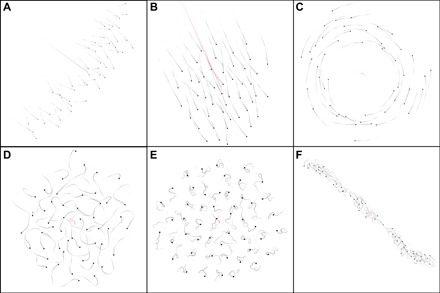
Different collective behaviors observed in the model for *N* = 50 individuals. Unless stated otherwise, $\alpha_{1}^{-1}=\beta_{1}^{-1}=12.5\text{BL}$. (**A**) Polarized on a line perpendicular to the movement (α_0_ = 0.2 and β_0_ = 0.01; movie S1). (**B**) Polarized in a circular shape (α_0_ = 0.5 and β_0_ = 0.1; movie S2). (**C**) Rotating. No preferred direction is chosen here, so individuals are turning in both directions at the same time (α_0_ = 0.1 and β_0_ = 0.02; movie S3). (**D**) Swarm behavior where individuals are moving freely in the swarm (α_0_ = 0.5 and β_0_ = 1; movie S4). (**E**) Crystal-like configuration (α_0_ = 0.1 and β_0_ = 10; movie S5). (**F**) Tube-like configuration ($\alpha_{1}^{-1}=\beta_{1}^{-1}=5\text{BL}$, α_0_ = 0.5, and β_0_ = 1; movie S6).

**Fig. 4:**
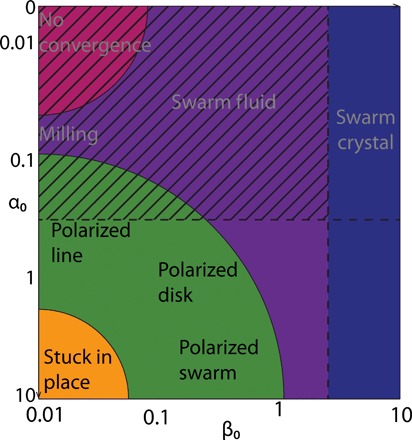
A qualitative phase diagram describing the modes of collective movement for the lowest-order vision-based model in the (α0,β0) parameter plane. In the zone with dashed lines, collisions are always observed. Outside of this zone, collisions can be avoided.

**Fig. 5:**
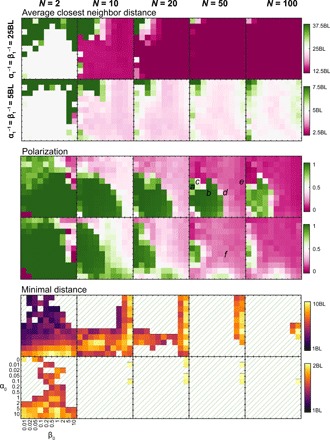
Results of the simulation. (**Top** to **bottom**) The average closest neighbor distance, the polarization of the swarm, and the minimal distance observed in the simulations (*42*) as function of α_0_ and β_0_. For different numbers of individuals (from left to right), *N* = 2, 10, 20, 50, and 100, and for two different values of the equilibrium distance, α_1_ = β_1_ = 25BL (top row) and α_1_ = β_1_ = 5BL (bottom row) (BL corresponds here to the diameter of the disk). For the minimal distance, dashed lines represent distances that are less than one BL, so the objects are colliding. Together with Fig. 4, those results can be used as a map to navigate the collection of video simulations of the model (*42*). The phase diagram gives a global overview, while this figure provides a more detailed, quantitative view on the system behavior. The letters *a*, *b*, *c*, *d*, *e*, and *f* indicate the parameters of the corresponding panels in Fig. 3.

A systematic exploration of the collective behavior of multiple agents interacting through the minimal vision model reveals the emergence of a wide range of collective behaviors for different parameter sets and group sizes (Figs. 3 to 5). In particular, we observe robust self-organized collective movements for a large range of parameters, emerging from the interplay of visual perception and the movement response of individuals. The degree of coordination and density of the flocks can be quantified through the normalized average velocity of the group, also referred to as orientational order or polarization, and the average nearest neighbor distance (Fig. 5). We note that because of the vision-induced long-ranged attraction, fragmentation of groups is negligible for the group sizes considered.

Figure 4 gives a qualitative overview over the location of different states in the (α_0_, β_0_) parameter plane, where α_0_ controls the overall acceleration-deceleration response and β_0_ controls the overall turning response. The exact boundary between the different regions depends on the number of individuals *N* and the equilibrium distance *L*_eq_. In Fig. 5, we show the corresponding quantitative results for neighbor distances and polarization values for different *N* and *L*_eq_.

Some general principles can be extracted. First, when both α_0_ and β_0_ are small, i.e., individuals have a very low overall response to the visual projection field, no polarization is observed, and the average closest neighbor distance is high. Obviously, in the limit of individuals not reacting to their neighbors, they will just move in straight lines, and the interindividual distance will naturally increase up to infinity. As long as either α_0_ or β_0_ is large enough, then the average distance to the closest neighbor decreases. Thus, individuals remain close to at least one other individual. This distance becomes smaller than *L*_eq_ when there are more than two individuals.

For α_0_ = 0, where individuals do not modify their acceleration, two transitions can be observed. First, as the turning rate β_0_ increases, the system reaches a swarm-like state, where individuals permanently change their positions within the group in a fluid-like manner (Fig. 3D). When β_0_ is increased even further, the system remains in a disordered state, but the average positions of individuals become locked in place (Fig. 3E), i.e., individuals move around fixed relative positions resulting in a crystal-like group structure.

On the other hand, when β_0_ is small, i.e., the individual turning response is small, three transitions are observed. First, as the linear acceleration rate α_0_ increases, the system reaches a swarm-like state where the position of the individuals remains fluid inside the group. In this parameter regime, milling states can be observed (Fig. 3C). We note that most of the time, no common rotation direction emerges, i.e., individuals turn in both directions simultaneously. When α_0_ is increased further, a polarized state is observed, where individuals arrange approximately along a line perpendicular to the direction of motion (Fig. 3A). This is close to the trivial steady state with individuals arranged in a perfect line, where, because of occlusions, an individual in the middle interacts only with its two closest neighbors to the left and right. The shape of the polarized group can be modified from this line state to an elliptical shape by increasing the turning rate β_0_ (Fig. 3B). If α_0_ is increased further while β_0_ remains low, the group gets stuck in place. Individuals are oscillating forth and back along their heading direction: They approach their neighbors but then move back when they come too close. Because of the erratic individual motion in this regime, no ordered steady state emerges. Note that those patterns are modified when the number of individuals is modified or when *L*_eq_ is changed (Fig. 4). In particular, groups with small numbers of individuals almost always display strong polarization.

Two mechanisms may explain the observed decrease in polarization with group size *N* (Fig. 5): First, because of occlusions, agents only perceive visual projections of a subset of the entire swarm, which may lead to decreasing global coordination with increasing *N*. Second and more likely, it is a consequence of the binary nature of the visual projection. With increasing group size, the visual projection becomes less and less informative because of increasing overlaps of projections from different individuals at different distances up to (partial) saturation of the visual field. This visual “confusion” inhibits the ability of the group to coordinate. The latter mechanism is also in line with smaller parameter regions where large, polarized groups can be observed (*P >* 0.5, *N* ≥ 10) for the smaller equilibrium distance *L*_eq_, resulting in higher flock densities (Fig. 5).

Last, for large groups, another collective mode becomes very prominent. The group assumes a tube-like geometry by spreading out in one spatial dimension, with individuals moving mainly along the main axis of the tube (Fig. 3E). This state can also be observed in smaller groups for small values of *L*_eq_.

Besides the ability to exhibit ordered, directed collective movement, an often neglected property is the ability of agents to avoid collisions. This might be particularly critically important for artificial swarm robotic systems. Here, we can identify extended regions of parameter space without any collisions overlapping with the regions of ordered motion (Figs. 4 and 5).

The observation of coordinated motion without any collisions is, in particular, remarkable, as our minimal vision model does not take any time derivatives of the visual field (i.e., optical flow) into account and thus lacks any explicit or implicit alignment mechanisms [e.g., see (*6*, *7*, *21*)]. Furthermore, individuals do not know where they are relative to others; thus, they do not use any information on the number or the distance of other individuals.

The absence of collision is observed in two main regions of the phase diagram (Fig. 5): when the turning rate is high (individuals swarm in a crystal-like configuration) and when the acceleration term is high. A balance needs to be found between acceleration rate and turning rate to generate noncolliding polarized swarm. Because of the symmetry of the interaction field, modifying linear acceleration is crucial for reliable avoidance of direct collisions. This emphasizes the importance of the individual speed modulation [c.f., (*20*)] and questions the generality of flocking systems where individuals move with constant speed and respond to others only through changes in their direction of motion. The ability to accelerate and decelerate is critical for obtaining noncolliding polarized swarm in the absence of explicit velocity alignment forces [see also (*22*)].

### Extension to 3D

**Fig. 6:**
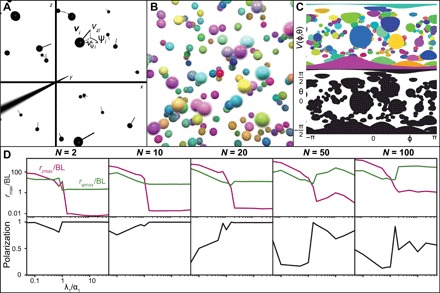
Collective movements in 3D. (**A**) A set of spheres with diameter BL is considered. Each sphere is propelled with a velocity *v_i_*(*t*) = *v*ψ*_i_*(*t*)*e*ψ(*t*) + *v_zi_*(*t*)*_ez_*. (**B**) A set of colored spheres is randomly distributed in space. The point of view of the red sphere is considered here with an idealized omnidirectional field pointing inside the image. (**C**) The projection of the visual field in 3D is given by a 2D surface. On top, objects can be represented by their colors. However, on the bottom part, a binary visual field is given. It is not possible to distinguish individuals. Because of the spherical nature of the projection, objects seem more deformed when they are further away from the horizon (θ = 0). (**D**) Top to bottom: The average closest neighbor distance and the polarization of the swarm and the minimal distance observed in the simulations (*42*) as function of λ_1_. For different numbers of individuals, from left to right, *N* = 2, 10, 20, 50, and 100. α_1_ = β_1_ = 0.1, α_0_ = 5.0, β_0_ = 2.0, and λ_0_ = 10.0.

Extending the model to three spatial dimensions can be performed in a straightforward way yet is not trivial. For this, we consider now the full visual projection in spherical coordinates for each individual by taking into account the corresponding azimuth angle of θ*_i_*. An additional equation is required to account for the variation of velocity in the third dimension. This could be implemented either with cylindrical coordinates, through the variation of velocity in the *z* direction *v_zi_* (Fig. 6A), or with spherical coordinates where the individuals are able to rotate in all spatial directions.

This modeling choice raises new fundamental questions related to kinematics, perception, and neural representation in the context of collective behavior. If the individuals can rotate in 3D, should the visual projection field be linked to the individual and thus be decoupled from the outside reference of left-right and up-down in the real world? Or should we rather assume an external reference frame, defined, e.g., via gravity, that anchors the visual projection so that the horizon remains horizontal? Here, recent insights from neuroscience may help resolve these questions, e.g., for bats, the existence of such a gravity-anchored reference frame has been recently suggested (*23*, *24*).

Furthermore, the question of the role of edge detection and response along different directions becomes conceptually nontrivial. Here, in a simple extension of the 2D case discussed above, we focus only on left-right edges with the derivative ∂_φ*i*_*V* and neglect, for simplicity, the impact of up-down edges, ∂_θ*i*_*V*.

These conceptual questions require a deeper analysis that is beyond the scope of this paper. The simple example discussed here is meant as a proof of concept that the minimal model can be extended to 3D and already yields potentially interesting dynamics for binary visual projections. Specifically, we make the following simplifying assumptions: Individuals can move in the *z* direction without rotation in θ and independently in the (*x,y*) plane. The visual field is thus always anchored to the real world. Derivatives are only considered in the left-right direction to be consistent with the analysis performed in 2D. The variation of velocity in the *z* direction is performed by comparing elements that are up and down. The variation of movement in the horizontal plane is only defined by the individuals that are contained in that plane. The corresponding equations of motion read

$$\begin{array}{cc} \partial_{t}v_{\psi i}(t)= & \int_{-\pi /2}^{\pi /2}d\theta_{i}\text{cos}\;\theta_{i}\int_{-\pi }^{\pi }d\phi_{i}\text{cos}\;\phi_{i}\alpha_{0}(-V_{i}(\phi_{i},\theta_{i},t)+\\ & \alpha_{1}{\left(\partial_{\phi_{i}}V_{i}(\phi_{i},\theta_{i},t)\right)}^{2})+\gamma (v_{0}-v_{i}(t)) \end{array}$$ (5)

$$\begin{array}{cc} \partial_{t}\psi_{i}(t)= & \int_{-\pi /2}^{\pi /2}d\theta_{i}\text{cos}\;\theta_{i}\int_{-\pi }^{\pi }d\phi_{i}\text{sin}\;\phi_{i}\beta_{0}(-V_{i}(\phi_{i},\theta_{i},t)+\\ & \beta_{1}{\left(\partial_{\phi_{i}}V_{i}(\phi_{i},\theta_{i},t)\right)}^{2}) \end{array}$$ (6)

$$\partial_{t}v_{\mathrm{zi}}(t)=\int_{-\pi /2}^{\pi /2}d\theta_{i}\text{sin}\;\theta_{i}\int_{-\pi }^{\pi }d\phi_{i}\lambda_{0}(-V_{i}(\phi_{i},\theta_{i},t)+\lambda_{1}{\left(\partial_{\phi_{i}}V_{i}(\phi_{i},\theta_{i},t)\right)}^{2})$$ (7)

We propose that in the *z* direction, the agents are still attracted to edges and repelled by angular area of the objects in their visual projection. From the pure point of view of symmetry of the system, it is then natural that the focal individual does not respond through vertical motion to objects in its horizontal plane. In other words, if all individuals are located in the horizontal plane, *z_i_* = 0 and θ*_i_* = 0, no movement direction in *z* (up or down) can be chosen unless a bias is introduced.

One needs to be careful that when objects are moving further away, their apparent size in the visual field is reduced not only along the ϕ axis but also along the θ axis [see Fig. 6 (B and C) for an impression of the visual field in 3D]. This leads to a situation where both the attraction and the repulsion strength decrease at infinity (see the Supplementary Materials for more details on the comparison between the 2D and 3D models). The attraction interaction toward the edges of objects acts now only at intermediate ranges. However, a linear correlation still exists between the equilibrium distances in the horizontal plane (*x,y*) and the values of $\alpha_{1}^{-1}$ and $\beta_{1}^{-1}$. It is expected that a similar equilibrium distance is given in the direction *z* by the inverse of the vertical edge attraction coefficient $\lambda_{1}^{-1}$.

We focus here only on the effect of the equilibrium distance in the *z* direction by setting the equilibrium distances equal in both directions in the plane, $\alpha_{1}^{-1}=\beta_{1}^{-1}=5\text{BL}$. We set α_0_ = 5 and β_0_ = 2 so that polarization is observed in the horizontal plane (*x,y*) when λ_0_ = 0. Last, we chose a large value for the vertical response parameter λ_0_ to reduce convergence time in the *z* direction: λ_0_ = 10. With these settings, we investigate simple metrics quantifying the shape and coordination of the swarm, namely, the maximal extension of the swarm in the plane (*x,y*), *r*_ψmax_, and in the direction *z*, *r*_*z*max_, as well as the average polarization of the swarm (Fig. 6D).

Looking at the extension of the swarm in the *z* direction, *r*_*z*max_, a sharp transition is observed. When λ_1_ *>* α_1_, the system remains mainly in the horizontal plane, *r*_*z*max_ *<* BL, while if λ_1_ *<* α_1_, then the swarm expands more in the *z* direction and *r*_*z*max_ reaches values higher than 10BL. This qualitative pattern is independent of the number of individuals.

This transition can be intuitively understood through the analysis of the two equilibrium distances: When λ_1_ *<* α_1_, the equilibrium distance in the *z* direction is small compared to the equilibrium distance in the horizontal plane. The swarm extends in the direction of the larger equilibrium distance, i.e., the group flattens and becomes quasi-2D. An analogous explanation can be given when λ_1_ *>* α_1_. Here, the constraints in the horizontal plane dominate. The system becomes effectively more compressed in the *x,y* directions. As the individuals come close together in *x* and *y*, they need to increase their distance in *z* and the group extends vertically.

As the swarm extends vertically, i.e., when λ_1_ is small, its extension in the horizontal plane is also increasing (Fig. 6D). Large extension in *z* results in the dilution of neighbors approximately in the same (*x,y*) plane as the focal individual. Because of the construction of the model, individuals respond strongest to others in the same horizontal plane. The stronger tendency of neighbors being located outside of the plane of the focal individual results in weaker overall visual attraction, which, in consequence, leads to an increase in the horizontal interindividual distance beyond the theoretical value *L*_eq_ obtained in the pure 2D case.

Last, for strongly anisotropic configurations, polarization seems to drop. In large swarms, polarization appears to become maximal when both equilibrium distances are of the same order of magnitude (λ_1_*/*α_1_ ≈ 1). For large *z* extensions, the reduced coordination can be explained again with the overall reduction in visual interactions due to non-negligible *z* differences between neighbors. For small extension in the *z* direction, we end up effectively with a 2D group. Here, the visual confusion in large groups due to binary visual projection, as discussed for the 2D case, leads to lower levels of coordination. Thus, more isotropic groups can be viewed as an optimal configuration of quasi-horizontal layers, which maximize in-plane coordination while minimizing off-plane dilution of visual interactions, resulting in maximal polarization of the entire group.

## DISCUSSION

The central aim of this work is the formulation of a mathematical framework for collective movement based exclusively on the visual projection field. Following a bottom-up approach, in this work, we focused on the simplest possible case: individual motion response to a binary visual projection field based on lowest-order expansion of the vision processing function *G*[*V*].

By relating perception and movement response, we have shown how a simple purely vision-based model of collective behavior can be constructed directly without the need for explicit ad hoc rules of coordination between individuals. This model does not specify spatial representation, explicit alignment, or even an explicit representation of other individuals. Therefore, these features are not essential ingredients of social interactions underlying organized collective behavior. It is important to emphasize this last point: The model cannot be simply rewritten and reformulated as a “classical” social-force model in the referential of an individual. Hence, it also calls into question the underlying representations implicitly assumed in social-force models.

Can animals identify the positions in space of other individuals? How many neighbors can be represented simultaneously from the vision of an individual? The answer to this question should arise from neurophysiological data, but their link to the movement response needs to be explicitly stated. Furthermore, a spatial scale is introduced in the system through the size of the animal and not through ad hoc parameters in the equation. The here-formulated mathematical model framework allows us to study the effects so far largely neglected in mathematical models of flocking, such as the role of the body shape of individuals in the visual projections or the role of coloration patterns on vision-mediated collective behaviors.

Eventually, we are convinced that a perception-based modeling framework will help build bridges between collective behavior research and sensory neuroscience (*4*). Specifically, a systematic bottom-up approach revealing discrepancies between predictions of minimal vision-based models and empirical observations will provide fundamental insights into the role of neural representations and higher-order processing of visual inputs in collective behavior.

In general, all theoretical models of flocking, including this one, should be critically assessed in terms of their relevance for real-world biological or artificial flocks. Despite the simplicity of our minimal model discussed above, we have shown that it can reproduce the social response map reported for pairs of fish (*20*) while, at the same time, producing coordinated movement patterns in larger groups of up to 100 agents. Nevertheless, even with this fundamental agreement, we should be cautious regarding the ability of this simple model to account for a broad range of collective behaviors observed in vertebrates. It relies only on lowest-order terms in the expansion of the visual response function *G*[*V*]; thus, it is likely more suited for describing coordination in scenarios where higher-order processing can be expected to play a very limited role, as in collective escape cascades in fish schools (*12*). Low-order, vision-based interactions are likely more relevant for collective behavior of invertebrates, such as insects [e.g., locusts (*25*) and midges (*26*)] or crustaceans [e.g., soldier crabs (*27*) and Antarctic krill (*28*)]. Here, juvenile locusts appear to be a promising study system, where we observe effective coordination and collective migration (*25*, *29*) of individuals with stereotypical optomotor responses and a still-developing, thus limited, visual system (*30*–*32*).

We note that the estimation of the visual projection field from video data requires more information than individual center of mass coordinates typically recorded in standard tracking experiments. It is essential to quantify the body shapes, head positions, and orientations. Whereas corresponding advanced tracking methods of animal groups are being actively researched (*33*), currently, available datasets lack this information. Hence, substantial additional effort is required to extract visual projection fields even for existing datasets, which goes beyond the scope of this work. In the future, a particularly promising avenue for investigating vision-based social interactions is state-of-the-art virtual reality techniques (*34*).

For minimal vision models with binary visual projection in the limit of very large and dense flocks, we may obtain full saturation of the visual field for individuals within the flock. In this case, the social response of individuals with saturated vision vanishes, whereas the individuals at the boundary experience a social attraction into the flock. Our results suggest that even in the absence of full saturation, overlaps in visual projection may inhibit coordination in large groups. These examples show the limitations of the minimal model relying on binary vision. However, we note that bird flocks operate at marginal opacity, without saturation of visual projection field, and the attraction toward edges in the visual projection in the minimal model is an effective mechanism for density regulation toward marginal opacity states (*15*). Furthermore, extremely high densities necessary for saturation of visual projection in bird flocks would require interindividual distances, which would make collisions very likely for empirically derived parameters (*5*). These extremely high densities are more likely to occur in large schools of pelagic fish, such as sardines or herring (*35*, *36*). The simplest solution for avoiding saturation of the visual projection field is to abandon the restriction of a binary visual projection. For example, for plainly colored schooling fish, neighbors can be assumed to blend with the background with increasing distance. A simple way to model decreasing contrast with distance is to assume a grayscale visual projection field where darkness decreases with distance. Thresholds in contrast detection would then naturally result in a visual response only to the first shells of nearest neighbors avoiding full saturation of the visual field. Ideally, corresponding distance dependencies and thresholds can be obtained from the properties of the visual system and/or the optical properties of the medium due to attenuation and scattering of light (*37*). Last but not least, for extremely high densities and corresponding short nearest-neighbor distances, other senses, such as touch and lateral line, will play an important role in movement coordination (*38*, *39*).

In contrast, at low density, an inaccurate visual system may fail to capture other individuals if they are too far away. Individuals would then become effectively invisible, and the interaction would vanish at infinity. Care needs to be taken when designing artificial systems to check that the size of the individuals and the expected size of the swarm can be captured by the used visual sensors.

Even if the simple model discussed above does not account for the full complexity of sensory and cognitive processing in humans or many vertebrates, we have demonstrated its ability to produce various modes of collective movement already with minimal assumptions on the vision-based interactions. Therefore, it represents an interesting reference model for self-organization of flocks, which is radically different from similar idealized models widely used in literature, such as the Vicsek model (*7*, *21*) or more biologically inspired models relying on phenomenological social forces (*1*–*3*).

We believe that the model framework is also of relevance to the theory of dynamical systems from a very fundamental point of view. It is a paradigmatic example of a class of models where interaction between individual units is not based on physical force fields but solely on the perception and internal representation of the social environment by the local agent. The coupling between agents is based on a lower-dimensional projection of the actual dynamical behavior of many agents. The resulting flocking model is neither metric nor topological (*11*); thus, new mathematical approaches are needed to explore the emergent collective behaviors at the macroscopic scale. Furthermore, the simple vision-only interaction discussed here has some interesting properties. It does not correspond to a simple superposition of binary interactions and does not rely on arbitrary cutoffs or thresholds. Thus, it results in a self-consistent description of interactions from a single individual up to large groups, naturally accounting for effects like self-organized marginal opacity (*15*) due to saturation of the visual field.

This vision-based model can also be useful for the construction of terrestrial and aerial robotics swarms. The ability to avoid collisions is given directly to each individual agent without the implementation of specific algorithms (*40*). Organized collective behavior can emerge from the instantaneous reaction to the visual projection field. The whole system is fully decentralized, and the collective organization does not rely on any explicit exchange of information between individuals. Once an omnidirectional, binary visual field is available, then the local computational requirements are low. The acquisition of a full field of view may pose a technical challenge, but the integrative nature of the model can be used efficiently. Expanding on works such as in (*41*), an array of sensors can perform independent computations and only exchange the results of the local integration. We show that the reduction of complex environmental perception through integration is sufficient for effective coordination. Minimal information bandwidth is then required between parts of the computational system. This final aspect reveals an interesting analogy to the perceptual modularity of our own brain, where the scene that we observe with both our eyes does not need to be fully exchanged between both sides of the brain.

## Supplementary Material

http://advances.sciencemag.org/cgi/content/full/6/6/eaay0792/DC1

Download PDF

Movie S1

Movie S2

Movie S3

Movie S4

Movie S5

Movie S6

A model of collective behavior based purely on vision

## SUPPLEMENTARY MATERIALS

Supplementary material for this article is available at http://advances.sciencemag.org/cgi/content/full/6/6/eaay0792/DC1 Fig. S1. Stable solution defined for the speeding force and the turning force. Fig. S2. Derivatives of simple discontinuous function. Fig. S3. Interaction strength in 2D and 3D. Fig. S4. Effects of the terms of Eq. 3 (main text) on a focal observer according to the relative position the other disk (blue). Movie S1. Dynamics observed in the model for *N* = 50 individuals [polarized on a line perpendicular to the movement ($\alpha_{1}^{-1}=\beta_{1}^{-1}=12.5\text{BL}$, α_0_ = 0.2, and β_0_ = 0.01)]. Movie S2. Dynamics observed in the model for *N* = 50 individuals [polarized in a circular shape ($\alpha_{1}^{-1}=\beta_{1}^{-1}=12.5\text{BL}$, α_0_ = 0.5, and β_0_ = 0.1)]. Movie S3. Dynamics observed in the model for *N* = 50 individuals [rotating; no preferred direction is chosen here, so individuals are turning in both directions at the same time ($\alpha_{1}^{-1}=\beta_{1}^{-1}=12.5\text{BL}$, α_0_ = 0.1, and β_0_ = 0.02)]. Movie S4. Dynamics observed in the model for *N* = 50 individuals [swarm behavior where individuals are moving freely in the swarm ($\alpha_{1}^{-1}=\beta_{1}^{-1}=12.5\text{BL}$, α_0_ = 0.5, and β_0_ = 1)]. Movie S5. Dynamics observed in the model for *N* = 50 individuals [crystal-like configuration ($\alpha_{1}^{-1}=\beta_{1}^{-1}=12.5\text{BL}$, α_0_ = 0.1, and β_0_ = 10)]. Movie S6. Dynamics observed in the model for *N* = 50 individuals [tube-like configuration ($\alpha_{1}^{-1}=\beta_{1}^{-1}=5\text{BL}$, α_0_ = 0.5, and β_0_ = 1)].

## Appendix

View/request a protocol for this paper from *Bio-protocol*.

## References

[1] Huth A., Wissel C., The simulation of the movement of fish schools. J. Theor. Biol. 156, 365–385 (1992).

[2] Couzin I. D., Krause J., James R., Ruxton G. D., Franks N. R., Collective memory and spatial sorting in animal groups. J. Theor. Biol. 218, 1–11 (2002). 12297066 10.1006/jtbi.2002.3065

[3] Couzin I. D., Krause J., Franks N. R., Levin S. A., Effective leadership and decision-making in animal groups on the move. Nature 433, 513–516 (2005). 15690039 10.1038/nature03236

[4] Lemasson B. H., Anderson J. J., Goodwin R. A., Collective motion in animal groups from a neurobiological perspective: The adaptive benefits of dynamic sensory loads and selective attention. J. Theor. Biol. 261, 501–510 (2009). 19699212 10.1016/j.jtbi.2009.08.013

[5] Hildenbrandt H., Carere C., Hemelrijk C. K., Self-organized aerial displays of thousands of starlings: A model. Behav. Ecol. 21, 1349–1359 (2010).

[6] Romanczuk P., Schimansky-Geier L., Swarming and pattern formation due to selective attraction and repulsion. Interface Focus 2, 746–756 (2012). 24312728 10.1098/rsfs.2012.0030 PMC3499126

[7] Vicsek T., Zafeiris A., Collective motion. Phys. Rep. 517, 71–140 (2012).

[8] Calovi D. S., Litchinko A., Lecheval V., Lopez U., Pérez-Escudero A., Chaté H., Sire C., Theraulaz G., Disentangling and modeling interactions in fish with burst-and-coast swimming reveal distinct alignment and attraction behaviors. PLOS Comput. Biol. 14, e1005933 (2018). 29324853 10.1371/journal.pcbi.1005933 PMC5783427

[9] Danjo T., Toyoizumi T., Fujisawa S., Spatial representations of self and other in the hippocampus. Science 359, 213–218 (2018). 29326273 10.1126/science.aao3898

[10] Omer D. B., Maimon S. R., Las L., Ulanovsky N., Social place-cells in the bat hippocampus. Science 359, 218–224 (2018). 29326274 10.1126/science.aao3474

[11] Strandburg-Peshkin A., Twomey C. R., Bode N. W., Kao A. B., Katz Y., Ioannou C. C., Rosenthal S. B., Torney C. J., Wu H. S., Levin S. A., Couzin I. D., Visual sensory networks and effective information transfer in animal groups. Curr. Biol. 23, R709–R711 (2013). 24028946 10.1016/j.cub.2013.07.059 PMC4780851

[12] Rosenthal S. B., Twomey C. R., Hartnett A. T., Wu H. S., Couzin I. D., Revealing the hidden networks of interaction in mobile animal groups allows prediction of complex behavioral contagion. Proc. Natl. Acad. Sci. U.S.A. 112, 4690–4695 (2015). 25825752 10.1073/pnas.1420068112 PMC4403201

[13] Collignon B., Séguret A., Halloy J., A stochastic vision-based model inspired by zebrafish collective behaviour in heterogeneous environments. R. Soc. Open Sci. 3, 150473 (2016). 26909173 10.1098/rsos.150473 PMC4736928

[14] Pita D., Collignon B., Halloy J., Fernández-Juricic E., Collective behaviour in vertebrates: A sensory perspective. R. Soc. Open Sci. 3, 160377 (2016). 28018616 10.1098/rsos.160377 PMC5180114

[15] Pearce D. J., Miller A. M., Rowlands G., Turner M. S., Role of projection in the control of bird flocks. Proc. Natl. Acad. Sci. U.S.A. 111, 10422–10426 (2014). 25002501 10.1073/pnas.1402202111 PMC4115545

[16] Moussaïd M., Helbing D., Theraulaz G., How simple rules determine pedestrian behavior and crowd disasters. Proc. Natl. Acad. Sci. U.S.A. 108, 6884–6888 (2011). 21502518 10.1073/pnas.1016507108 PMC3084058

[17] Lavergne F. A., Wendehenne H., Bäuerle T., Bechinger C., Group formation and cohesion of active particles with visual perception–dependent motility. Science 364, 70–74 (2019). 30948548 10.1126/science.aau5347

[18] F. Schilling, J. Lecoeur, F. Schiano, D. Floreano, Learning vision-based cohesive flight in drone swarms. arXiv: 1809.00543 [cs.RO] (2018).

[19] Barberis L., Peruani F., Large-scale patterns in a minimal cognitive flocking model: Incidental leaders, nematic patterns, and aggregates. Phys. Rev. Lett. 117, 248001 (2016). 28009185 10.1103/PhysRevLett.117.248001

[20] Katz Y., Tunstrøm K., Ioannou C. C., Huepe C., Couzin I. D., Inferring the structure and dynamics of interactions in schooling fish. Proc. Natl. Acad. Sci. U.S.A. 108, 18720–18725 (2011). 21795604 10.1073/pnas.1107583108 PMC3219116

[21] Chaté H., Ginelli F., Grégoire G., Peruani F., Raynaud F., Modeling collective motion: Variations on the vicsek model. Eur. Phys. J. B 64, 451–456 (2008).

[22] Romanczuk P., Couzin I. D., Schimansky-Geier L., Collective motion due to individual escape and pursuit response. Phys. Rev. Lett. 102, 010602 (2009). 19257176 10.1103/PhysRevLett.102.010602

[23] Finkelstein A., Derdikman D., Rubin A., Foerster J. N., Las L., Ulanovsky N., Three-dimensional head-direction coding in the bat brain. Nature 517, 159–164 (2015). 25470055 10.1038/nature14031

[24] D. E. Angelaki, J. Ng, A. M. Abrego, H. X. Cham, J. Dickman, J. Laurens, A gravity-based three-dimensional compass in the mouse brain. bioRxiv 570382 [Preprint]. 2019. 10.1038/s41467-020-15566-5 PMC7160108 32296057

[25] Buhl J., Sumpter D. J., Couzin I. D., Hale J. J., Despland E., Miller E. R., Simpson S. J., From disorder to order in marching locusts. Science 312, 1402–1406 (2006). 16741126 10.1126/science.1125142

[26] Attanasi A., Cavagna A., Del Castello L., Giardina I., Melillo S., Parisi L., Pohl O., Rossaro B., Shen E., Silvestri E., Viale M., Collective behaviour without collective order in wild swarms of midges. PLOS Comput. Biol. 10, e1003697 (2014). 25057853 10.1371/journal.pcbi.1003697 PMC4109845

[27] Murakami H., Tomaru T., Niizato T., Nishiyama Y., Sonoda K., Moriyama T., Gunji Y.-P., Collective behavior of soldier crab swarm in both ring- and round-shaped arenas. Artif. Life Robot. 20, 315–319 (2015).

[28] Kawaguchi S., King R., Meijers R., Osborn J. E., Swadling K. M., Ritz D. A., Nicol S., An experimental aquarium for observing the schooling behaviour of antarctic krill (euphausia superba). Deep-Sea Res. II Top. Stud. Oceanogr. 57, 683–692 (2010).

[29] Bazazi S., Romanczuk P., Thomas S., Schimansky-Geier L., Hale J. J., Miller G. A., Sword G. A., Simpson S. J., Couzin I. D., Nutritional state and collective motion: From individuals to mass migration. Proc. Biol. Sci. 278, 356–363 (2010). 20739320 10.1098/rspb.2010.1447 PMC3013415

[30] Wallace G., Some experiments on form perception in the nymphs of the desert locust, *Schistocerca gregaria* Forskål. J. Exp. Biol. 35, 765–775 (1958).

[31] Eley S., Shelton P. M. J., Cell junctions in the developing compound eye of the desert locust *Schistocerca gregaria*. Development 36, 409–423 (1976). 1003079

[32] M. Burrows, *The Neurobiology Of An Insect Brain* (Oxford Univ. Press on Demand, 1996).

[33] Graving J. M., Chae D., Naik H., Li L., Koger B., Costelloe B. R., Couzin I. D., Deepposekit, a software toolkit for fast and robust animal pose estimation using deep learning. eLife 8, e47994 (2019). 31570119 10.7554/eLife.47994 PMC6897514

[34] Stowers J. R., Hofbauer M., Bastien R., Griessner J., Higgins P., Farooqui S., Fischer R. M., Nowikovsky K., Haubensak W., Couzin I. D., Tessmar-Raible K., Straw A. D., Virtual reality for freely moving animals. Nat. Methods 14, 995–1002 (2017). 28825703 10.1038/nmeth.4399 PMC6485657

[35] Pitcher T., Partridge B., Fish school density and volume. Mar. Biol. 54, 383–394 (1979).

[36] Handegard N. O., Boswell K. M., Ioannou C. C., Leblanc S. P., Tjøstheim D. B., Couzin I. D., The dynamics of coordinated group hunting and collective information transfer among schooling prey. Curr. Biol. 22, 1213–1217 (2012). 22683262 10.1016/j.cub.2012.04.050

[37] T. W. Cronin, S. Johnsen, N. J. Marshall, E. J. Warrant, *Visual Ecology* (Princeton Univ. Press, 2014).

[38] Partridge B. L., Pitcher T. J., The sensory basis of fish schools: Relative roles of lateral line and vision. J. Comp. Physiol. 135, 315–325 (1980).

[39] Bazazi S., Buhl J., Hale J. J., Anstey M. L., Sword G. A., Simpson S. J., Couzin I. D., Collective motion and cannibalism in locust migratory bands. Curr. Biol. 18, 735–739 (2008). 18472424 10.1016/j.cub.2008.04.035

[40] Dentler J., Rosalie M., Danoy G., Bouvry P., Kannan S., Olivares-Mendez M. A., Voos H., Collision avoidance effects on the mobility of a uav swarm using chaotic ant colony with model predictive control. J. Intell. Robot. Syst. 93, 227–243 (2019).

[41] C. Moeslinger, T. Schmickl, K. Crailsheim, *European Conference on Artificial Life* (Springer, 2009), pp. 375–382.

[42] R. Bastien, P. Romanczuk, P. Simulations (2019); http://unred.org/visualmodel, [accessed 23 March 2019].

