# Supplementary material for: A model of collective behavior based purely on vision
Source: Sci Adv. 2020 Feb 5;6(6):eaay0792. doi: 10.1126/sciadv.aay0792 (PMC7002123; doi:10.1126/sciadv.aay0792)
Supplement: http://advances.sciencemag.org/cgi/content/full/6/6/eaay0792/DC1 [file supp_6_6_eaay0792__index.html]

Science Advances | Science AdvancesAAASSearchScience AdvancesMenu

## Supplementary Materials

**The PDFset includes:**

- Fig. S1. Stable solution defined for the speeding force and the turning force.
- Fig. S2. Derivatives of simple discontinuous function.
- Fig. S3. Interaction strength in 2D and 3D.
- Fig. S4. Effects of the terms of Eq. 3 (main text) on a focal observer according to the relative position the other disk (blue).

Download PDF

**Other Supplementary Material for this manuscript includes the following:**

- Movie S1 (.mp4 format). Dynamics observed in the model for *N* = 50 individuals polarized on a line perpendicular to the movement ( α1−1=β1−1=12.5BL, α0 = 0.2, and β0 = 0.01).
- Movie S2 (.mp4 format). Dynamics observed in the model for *N* = 50 individuals polarized in a circular shape ( α1−1=β1−1=12.5BL, α0 = 0.5, and β0 = 0.1).
- Movie S3 (.mp4 format). Dynamics observed in the model for *N* = 50 individuals rotating; no preferred direction is chosen here, so individuals are turning in both directions at the same time ( α1−1=β1−1=12.5BL, α0 = 0.1, and β0 = 0.02).
- Movie S4 (.mp4 format). Dynamics observed in the model for *N* = 50 individuals swarm behavior where individuals are moving freely in the swarm ( α1−1=β1−1=12.5BL, α0 = 0.5, and β0 = 1).
- Movie S5 (.mp4 format). Dynamics observed in the model for *N* = 50 individuals crystal-like configuration ( α1−1=β1−1=12.5BL, α0 = 0.1, and β0 = 10).
- Movie S6 (.mp4 format). Dynamics observed in the model for *N* = 50 individuals tube-like configuration ( α1−1=β1−1=5BL, α0 = 0.5, and β0 = 1).

**Files in this Data Supplement:**

- Adobe PDF - aay0792\_SM.pdf
