# Supplementary material for: A model of collective behavior based purely on vision
Source: Sci Adv. 2020 Feb 5;6(6):eaay0792. doi: 10.1126/sciadv.aay0792 (PMC7002123; doi:10.1126/sciadv.aay0792)
Supplement: Download PDF [file aay0792_SM.pdf]

Science Advances
AAAS

advances.sciencemag.org/cgi/content/full/6/6/eaay0792/DC1

Supplementary Materials for

A model of collective behavior based purely on vision

Renaud Bastien* and Pawel Romanczuk

*Corresponding author. Email: renaud@unred.org

Published 5 February 2020, *Sci. Adv.* **6**, eaay0792 (2020)
DOI: 10.1126/sciadv.aay0792

The PDF file includes:

Fig. S1. Stable solution defined for the speeding force and the turning force.
Fig. S2. Derivatives of simple discontinuous function.
Fig. S3. Interaction strength in 2D and 3D.
Fig. S4. Effects of the terms of Eq. 3 (main text) on a focal observer according to the relative position the other disk (blue).

Other Supplementary Material for this manuscript includes the following:

(available at advances.sciencemag.org/cgi/content/full/6/6/eaay0792/DC1)

Movie S1 (.mp4 format). Dynamics observed in the model for *N* = 50 individuals [polarized on a line perpendicular to the movement ($\alpha_1^{-1} = \beta_1^{-1} = 12.5\text{BL}$, $\alpha_0 = 0.2$, and $\beta_0 = 0.01$)].
Movie S2 (.mp4 format). Dynamics observed in the model for *N* = 50 individuals [polarized in a circular shape ($\alpha_1^{-1} = \beta_1^{-1} = 12.5\text{BL}$, $\alpha_0 = 0.5$, and $\beta_0 = 0.1$)].
Movie S3 (.mp4 format). Dynamics observed in the model for *N* = 50 individuals [rotating; no preferred direction is chosen here, so individuals are turning in both directions at the same time ($\alpha_1^{-1} = \beta_1^{-1} = 12.5\text{BL}$, $\alpha_0 = 0.1$, and $\beta_0 = 0.02$)].
Movie S4 (.mp4 format). Dynamics observed in the model for *N* = 50 individuals [swarm behavior where individuals are moving freely in the swarm ($\alpha_1^{-1} = \beta_1^{-1} = 12.5\text{BL}$, $\alpha_0 = 0.5$, and $\beta_0 = 1$)].
Movie S5 (.mp4 format). Dynamics observed in the model for *N* = 50 individuals [crystal-like configuration ($\alpha_1^{-1} = \beta_1^{-1} = 12.5\text{BL}$, $\alpha_0 = 0.1$, and $\beta_0 = 10$)].
Movie S6 (.mp4 format). Dynamics observed in the model for *N* = 50 individuals [tube-like configuration ($\alpha_1^{-1} = \beta_1^{-1} = 5\text{BL}$, $\alpha_0 = 0.5$, and $\beta_0 = 1$)].

Model Construction

We start with equation 1. To simplify the calculations, we consider first the two-dimensional case and assume that the movement is restricted to a single plane. The velocity of an individual i, $\mathbf{v_i}$, is then described by its direction ψ_i and its magnitude v_i.

$$\mathbf{v_i} \quad = v_i \quad \begin{pmatrix} \cos\psi_i \\ \sin\psi_i \end{pmatrix} \tag{S1}$$

It is then possible to easily define elementary vectors relative to the orientation of the individual i

$$\mathbf{e}_v^i = \begin{pmatrix} \cos\psi_i \\ \sin\psi_i \end{pmatrix}, \quad \mathbf{e}_\psi^i = \begin{pmatrix} -\sin\psi_i \\ \cos\psi_i \end{pmatrix} \tag{S2}$$

so that

$$\mathbf{v_i} = v_i \mathbf{e}_v^i \tag{S3}$$

The equation 1 can be split in two equations

$$\partial_t v_i = \mathbf{F}^i_{ind} \cdot \mathbf{e}^i_v + \mathbf{F}^i_{vis}[V_i(\phi_i, \theta_i, t)] \cdot \mathbf{e}^i_v \tag{S4}$$

$$\partial_t \psi_i = \mathbf{F}^i_{ind} \cdot \mathbf{e}^i_\psi + \mathbf{F}^i_{vis}[V_i(\phi_i, \theta_i, t)] \cdot \mathbf{e}^i_\psi \tag{S5}$$

that describes the variation of magnitude u and the variation of direction respectively. Now that the basics have been defined, the model and its hypotheses are described. First, as the individual movement behavior is not the focus of this work, we assume the individual force $\mathbf{F_{ind}}$ to be defined by a simple linear friction/propulsion function

$$\mathbf{F}^i_{ind} = \gamma(v_0 - v_i)\mathbf{e}^i_v \tag{S6}$$

Here γ is a constant defining the relaxation rate of the individual velocity to the preferred velocity v_0. In addition we assume that there is no global preferred direction of motion, thus the velocity vector of the individual does not depend directly on ϕ. Eventually, $\mathbf{F}^i_{vis}$ can be rewritten as an integral over the visual space

$$\mathbf{F}_{\text{vis}}[V] = \int_{-\pi}^{\pi} d\phi_i G[V(\phi_i, t)]\mathbf{h}(\phi_i) \tag{S7}$$

with $\mathbf{h}(\phi)$ being an arbitrary vector function, determining the projection of $G[V]$ on the low-dimensional movement response. It can be seen as a target function for the visual input. The symmetries of the system can be inferred from (19). Here, they measure the values of $\partial_t v_i$ and $\partial_t \phi_i$ for one individual, as a function of the relative position of another individual. $\partial_t \phi_i$ is anti-symmetric between left and right whereas $\partial_t v_i$ is anti-symmetric between front and back. The target functions $\mathbf{h}(\phi)$ carries those symmetries and can be rewritten as

$$\mathbf{h}(\phi) = \sum_p a_p \cos(p\phi)\mathbf{e}_y + b_p \sin(p\phi)\mathbf{e}_\psi \tag{S8}$$

Furthermore, the assumption of $\mathbf{h}(\phi)$ component along $\mathbf{e}_v$ and $\mathbf{e}_\psi$ being symmetric and anti-symmetric functions around $\phi = 0$, respectively, is required to ensure the absence of permanent rotational motion of individual agents.

Inserting Eq. S8 into Eq. S7 eventually yields, the movement equations

$$\partial_t v_i = \sum_p \int_{-\pi}^{\pi} d_\phi a_p \cos(p\phi) G^S[V] + \gamma(v_0 - v_i) \tag{S9}$$

$$\partial_t \psi_i = \sum_p \int_{-\pi}^{\pi} d_\phi b_p \sin(p\phi) G^{AS}[V] \tag{S10}$$

Here, we split the function $G[V]$ into its symmetrical part, $G^S[V]$, and its anti-symmetrical part $G^{AS}[V]$,

$$G[V] = G^S[V] + G^{AS}[V] \tag{S11}$$

based on the non-zero contribution to the above integrals. The movement is then driven by the discrepancies in the symmetry of the visual field. The asymmetry between left and right will modify the direction of the individual while the asymmetry between front and back will modify the magnitude of the velocity.

Now we can rewrite the symmetric and anti-symmetric parts in terms of derivatives in time, $\partial_t^j V$, and space, $\partial_\phi^k V$

$$G^S[V] = \sum_{jkl} \alpha_{jkl} (\partial_t^j \partial_\phi^k V)^l \tag{S12}$$

$$G^{AS}[V] = \sum_{jkl} \beta_{jkl} (\partial_t^j \partial_\phi^k V)^l \tag{S13}$$

Until now we have been as general as possible. The visual field remains here an abstract function that needs to be appropriately defined. A tremendous diversity of parameters and orders can be considered. However, as we now have described the general model, it is possible to study each model independently, starting with the simplest one, and to extract the mechanisms expected at each scale. The simplest form of the the visual projection field is then obtained with the following assumptions

A1 The visual field is binary when an object is in the field $V = 1$, and if not $V = 0$ (i.e. no implicit relative distance information based e.g. on contrast, no color vision etc.),

A2 There is no higher-order cognitive processing by individuals (no individual identification, no selective attention, no hierarchy of individuals etc.).

If we only consider the first orders of the Fourier transform, the full model for a binary visual field is given by

$$\partial_t v_i(t) = \int_{-\pi}^{\pi} d\phi_i \cos\phi_i \alpha_0 \left(V_i(\phi_i,t) + \alpha_1 \left(\partial_s V_i(\phi_i,t)\right)^2 + \alpha_2 \partial_t V_i(\phi_i,t)\right) \tag{S14}$$

$$\partial_t \psi_i(t) = \int_{-\pi}^{\pi} d\phi_i \sin\phi_i \beta_0 \left(V_i(\phi_i,t) + \beta_1 \left(\partial_s V_i(\phi_i,t)\right)^2 + \beta_2 \partial_t V_i(\phi_i,t)\right) \tag{S15}$$

As the system remains invariant when flipping $\phi \to -\phi$, the square of the spatial derivative should be considered. Also, due to the binary nature of the visual field, higher order, and cross, derivatives do not contain more information. Similarly, rising the parameters of the visual field to a higher exponent yields the same function. Finally, we neglect the temporal derivatives, and consider only interactions based on the instantaneous visual projection.

Stable position

In order to understand which positions two individuals assume in relation to each other, it is important to define the transition between the effective repulsion and attraction, i.e. the stable state of system (fixed point of the dynamics). The relative position, d, of two individuals, f_i and f_j, respective to each other, is conserved if they are moving in the same direction $\phi_i = \phi_j$, with the same speed $v_i = v_j$. It comes trivially that $\partial_t v_i = 0$ if $\phi = k\pi + \pi/2$ with k integer and that $\partial_t \psi_i = 0$ if $\phi = k\pi$. Other solutions are then defined by

$$\partial_t v_i = \int_{-\pi}^{\pi} d\phi \cos\phi \alpha_0 \left(-V + \alpha_1 \partial_\phi V\right) = 0 \tag{S16}$$

$$\partial_t \psi_i = \int_{-\pi}^{\pi} d\phi \sin\phi \beta_0 \left(-V + \beta_1 \partial_\phi V\right) = 0 \tag{S17}$$

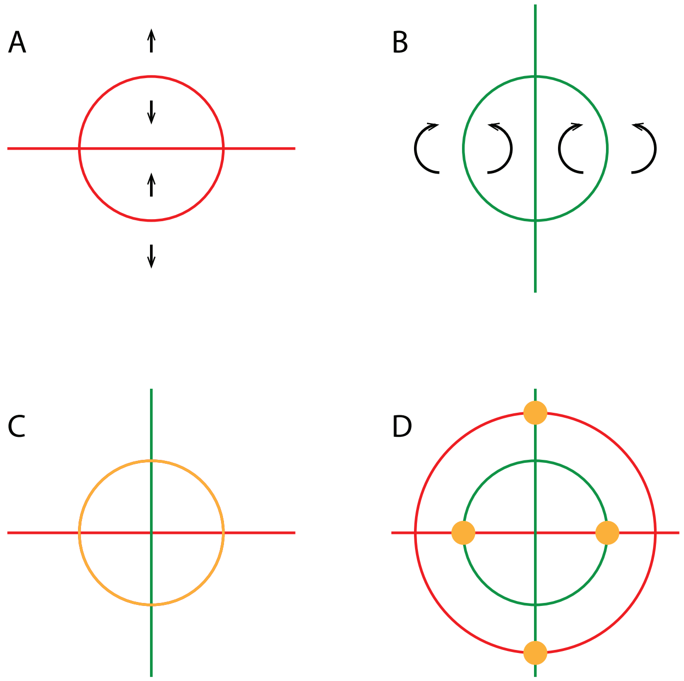

Fig. S1. Stable solution defined for the speeding force in red (A) and the turning force in green (B). Each stable solution is defined by the combination of a line and a circle. The solutions inside the circle are unstable, and only the solutions on the circle are stable. C. If the distance of the stable circle is the same for the turning force and the speeding force a ring of stable solutions is defined (in orange). If both distances are different, there exist 5 stable positions (in orange + the center of the circle) but only the two solutions the further from the center are stable.

For a pairwise interaction this becomes

$$\int_{\phi-\arctan\frac{R}{d}}^{\phi+\arctan\frac{R}{d}} d\phi\cos\phi = \alpha_1\left(\cos\left(\phi-\arctan\frac{R}{d}\right)+\cos\left(\phi+\arctan\frac{R}{d}\right)\right) \quad \text{(S18)}$$

$$\int_{\phi-\arctan\frac{R}{d}}^{\phi+\arctan\frac{R}{d}} d\phi\sin\phi = \beta_1\left(\sin\left(\phi-\arctan\frac{R}{d}\right)+\sin\left(\phi+\arctan\frac{R}{d}\right)\right) \quad \text{(S19)}$$

and

$$\left(\sin\left(\phi+\arctan\frac{R}{d}\right)-\sin\left(\phi-\arctan\frac{R}{d}\right)\right) = \alpha_1\left(\cos\left(\phi-\arctan\frac{R}{d}\right)+\cos\left(\phi+\arctan\frac{R}{d}\right)\right) \quad \text{(S20)}$$

$$\left(-\cos\left(\phi+\arctan\frac{R}{d}\right)+\cos\left(\phi-\arctan\frac{R}{d}\right)\right) = \beta_1\left(\sin\left(\phi-\arctan\frac{R}{d}\right)+\sin\left(\phi+\arctan\frac{R}{d}\right)\right) \quad \text{(S21)}$$

which can be simplified as

$$\cos\phi\sin\arctan\frac{R}{d} = \alpha_1\cos(\phi)\cos\left(\arctan\frac{R}{d}\right) \quad \text{(S22)}$$

$$\sin\phi\cos\arctan\frac{R}{d} = \beta_1\sin(\phi)\sin\left(\arctan\frac{R}{d}\right) \quad \text{(S23)}$$

and finally

$$d = R\alpha_1^{-1} \quad \text{(S24)}$$

$$d = R\beta_1^{-1} \quad \text{(S25)}$$

The solution defines a circle of radius $R\alpha_1^{-1}$ around the individual (fig. S1.A and B). In order to get repulsion for more than one body length $1 > \alpha_1^{-1}$ and $1 > \beta_1^{-1}$. If both lengths are similar a stable position is defined on a ring around the individual (Figure SS1.C). The solution being on a circle, the position is stable for both individual and a stable polarization state should be possible. If both lengths are different, only two stable positions are then found, $\phi = n\pi/2$. A line where each individual is moving perpendicularly to the main axis of the swarm has been easily observed in the simulation.

Numerical Simulations

A series of numerical simulations has been realized for

$$N = 2, 3, 4, 5, 6, 7, 8, 9, 10, 20, 50, 100, 200, 500.$$

Only a subset of this simulation is discussed here ($N = 2, 10, 20, 50, 100$), but videos of the emergent behavior can be viewed at (21). Each individual agents is a disk of radius $R = 0.5$, self-propelled with a velocity $v = 1$, and the speed relaxation rate is $\gamma = 0.1$. The time step between two updates of the velocity is $\Delta t = 0.1$ while the whole simulation runs until a time $T_{end} = 1000$. In all simulations $\alpha_1 = \beta_1$ so that the equilibrium distance in the front-back and left right direction are the same. Two different values are used so that $\alpha_1 = \beta_1 = 0.1$ and 0.02 accounting for equilibrium distance 5 and 25. For each set of parameters, range of values of α_0 and β_0 have been explored to study the influence of different sensitivity to acceleration and turning rate spanning multiple order of magnitude,

$$\alpha_0 = 0, 0.01, 0.02, 0.05, 0.1, 0.2, 0.5, 1, 2, 5, 10\ ,$$

and

$$\beta_0 = 0.01, 0.02, 0.05, 0.1, 0.2, 0.5, 1, 2, 5, 10$$

Each sets of parameters is then replicated 5 times so that a total of 13200 simulations has been performed.

Due to the simple vision-based interaction considered here, it was possible to avoid techniques such as ray-casting. Instead the visual projection field of each individual, V_i, is considered as a linear function of ϕ, from $-\pi$ to π, constituted of 16384 elements, so that $\Delta\phi = \pi/8192$. $V_i(\phi)$ is initialized at 0 for all ϕ at each time step. The visual field is then updated by considering the relative position of each individual j, in the referential of the focal individual i

oriented in the direction of the movement ψ_i, (x^i_j, y^i_j). The position in the visual field is recovered by computing the relative angle of each individual $\phi^i_j = \arctan y^i_j/x^i_j$, with the extension $\Delta\phi^i_j = \arctan R/d^i_j$, where $d^j_i = \sqrt{{x^i_j}^2 + {y^i_j}^2}$. The values of the visual projection field are the updated so that $V_i(\phi) = 1$ in the range $(\phi^i_j - \Delta\phi^i_j, \phi^i_j + \Delta\phi^i_j)$. As the visual field is binary, there is no need to considered supplementary calculation to account for occlusion. The velocity and turning rate of each individual at time $t + \Delta t$ is then updated according to its perceived visual projection

$$v_i(t+\Delta t) = v_i(t) + \Delta t \sum_{\phi=-\pi}^{\pi} \cos\phi\alpha_0\left(V\Delta\phi + \alpha_1\partial_\phi V\right) \tag{S26}$$

$$\psi_i(t+\Delta t) = \psi_i(t) + \Delta t \sum_{\phi=-\pi}^{\pi} \sin\phi\beta_0\left(V\Delta\phi + \beta_1\partial_\phi V\right) \tag{S27}$$

The minimal distance observed, d_{min} is measured by taking the minimal inter-individual distance at each time step and updating for all the time of the simulation

$$d_{min} = \min_{t=0}^{t=t_{end}}\left(\min_{i=1,j=1,i\neq j}^{i=N,j=N} d^j_i(t)\right) \tag{S28}$$

If $d_{min} < 1$, then collisions are observed and the computation is stopped.

The mean polarization p is measured as the average polarization between all individuals at each time step, averaged on the last 10% of each simulation, so in the temporal range $t = (900, 1000)$,

$$p = \frac{1}{100}\sum_{t=900}^{1000}\left(\frac{1}{\sum_{k=1}^{k=N-1} k}\sum_{i=1}^{i=N}\sum_{j=i,j\neq i}^{j=N}\frac{\mathbf{v_i}(t)\mathbf{v_j}(t)}{v_i(t)v_j(t)}\right) \tag{S29}$$

Finally the average closest neighbor distance is measured, d_{mean} as the average 10% of each simulation, so in the temporal range $t = (900, 1000)$, of the minimal inter-individual distance observed at each time step

$$d_{mean} = \frac{1}{100}\sum_{t=900}^{1000}\left(\min_{i=1,j=1,i\neq j}^{i=N,j=N} d^j_i(t)\right) \tag{S30}$$

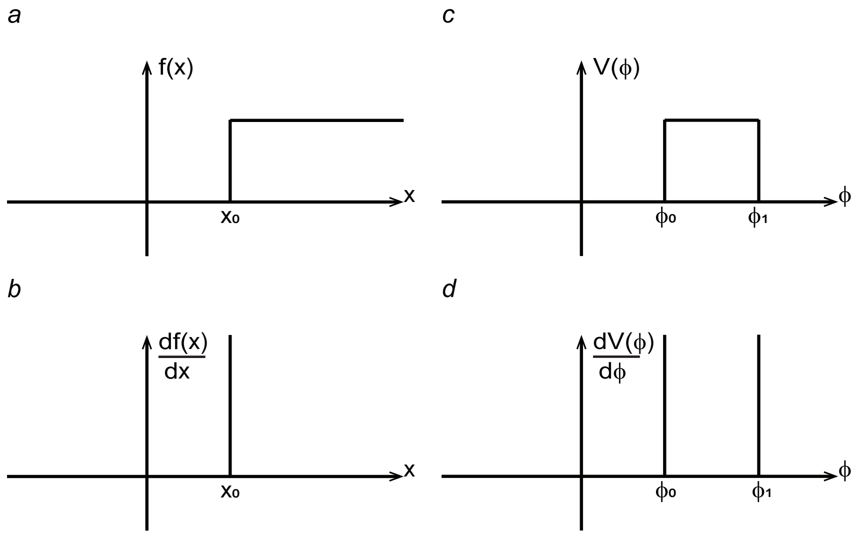

Fig. S2. **Derivatives of simple discontinuous function.** a. A Heavyside step function $f(x) = \mathcal{H}(x - x_0)$. b. The derivative of $f(x)$ is a Dirac delta function $df(x)/dx = \delta(x - x_0)$

Derivation of non-continuous function: the Dirac delta function

A binary visual field is by essence discontinuous. The edge of an object is localized on a point but does not extend spatially. The derivative of the visual field is infinite at those singular points, where the edges are located. This could be seen as an issue to solve the model properly. Fortunately, the theory of distributions has been introduced to give mathematical groundings to this class of problems. We do not plan to give here a complete mathematical review of distributions, and refer the reader to corresponding text books. Many textbooks in theoretical physics provide introductory chapters to the topic, specifically to discuss the properties the so-called Dirac delta function relevant here. Here, we give only a brief demonstration on how the Dirac delta function is used to solve the problem of derivation of non-continuous functions, so that non-specialists readers can appreciate the details of the model.

We start with a simple function $f(x)$ with a single point of discontinuity (fig. S2). It is then convenient to consider the Heavyside function $\mathcal{H}(x)$, also known as the unit step function

and defined as

$$\mathcal{H}(x) = \begin{cases} 0 & x < 0 \\ 1 & x \geq 0 \end{cases} \tag{S31}$$

We can now define $f(x)$ as

$$f(x) = \mathcal{H}(x - x_0) \tag{S32}$$

the point of discontinuity can then be defined at any position x_0 (Figure S2a). Formally, the derivative of the Heavyside function is given by the Dirac delta function $\delta(x)$, so that

$$\frac{d\mathcal{H}(x - x_0)}{dx} = \delta(x - x_0) \tag{S33}$$

The Dirac delta function is defined by

$$\delta(x) = \begin{cases} \infty & x = 0 \\ 0 & x \neq 0 \end{cases} \tag{S34}$$

This function is equal to 0 everywhere, expect at the point of the discontinuity (fig. S2b). A problem of convergence seems to arise at this discontinuity, the derivative is now infinite. Nonetheless, the integral of the Dirac delta function exists and is finite

$$\int_{-\infty}^{\infty} \delta(x) dx = 1 \tag{S35}$$

This brings an interesting property when calculating the integral of an arbitrary continuous function, $g(x)$, multiplied by a Dirac function

$$\int_{-\infty}^{\infty} \delta(x - x_0) g(x) dx = g(x_0) \tag{S36}$$

The result of the integral is then the value of the function $g(x)$ at the discontinuities of the Dirac function, *i.e.* x_0.

Going back to the visual field, a binary visual field can be explicitly defined as a series of Heavyside function. For instance, we consider a single object visible in the visual field, $V(\phi)$, that can be seen between the angle ϕ_0 and ϕ_1 (Figure S2c). This visual field is defined by

$$V(\phi) = \begin{cases} 1 & \phi_0 < \phi < \phi_1 \\ 0 & \text{otherwise} \end{cases} \tag{S37}$$

This equation can be rewritten in terms of Heaviside functions

$$V(\phi) = \mathcal{H}(\phi - \phi_0) + \mathcal{H}(-\phi + \phi_1) - 1 \quad \text{(S38)}$$

The derivative of the visual can then be easily computed with equation S35 (Figure S2d)

$$\frac{dV\phi}{d\phi} = \delta(\phi - \phi_0) - \delta(\phi - \phi_1) \quad \text{(S39)}$$

We now have all the elements necessary to compute the contribution of the derivative of the visual field on the movements of an individual. If we consider the term associated with the rotation of an individual in equation 1, we need to solve

$$\int_{-\pi}^{\pi} \left(\frac{dV\phi}{d\phi}\right)^2 \sin\phi d\phi = \int_{-\pi}^{\pi} \left(\delta(\phi - \phi_0) - \delta(\phi - \phi_1)\right)^2 \sin\phi d\phi \quad \text{(S40)}$$

Considering that

$$\delta(x - x_0)\delta(x - x_1) = \begin{cases} \delta(x - x_0) & x_0 = x_1 \\ 0 & x_0 \neq x_1 \end{cases} \quad \text{(S41)}$$

it comes directly that

$$\int_{-\pi}^{\pi} \left(\frac{dV\phi}{d\phi}\right)^2 \sin\phi d\phi = \sin\phi_0 + \sin\phi_1 \quad \text{(S42)}$$

This can then be extended to any number of discontinuities in the visual field.

Extension to 3D

When extending to 3D, one needs to be careful that the size of the object is going to be reduced in two directions at the same time. If we consider the influence of an object that is just in front of an observer on the acceleration in the plane, $\partial_t v_{\psi i}$, there are differences visible between 2D and 3D (Figure S3). The influence due to the subtended angle is higher in 3D than in 2D when the object is close. But the decay is faster as the object is moving further away. The influence of the edges is also higher when the object is close, but the influence of far-away object is decaying to infinity. If we calculate analytically the equilibrium distance at which the interaction switches

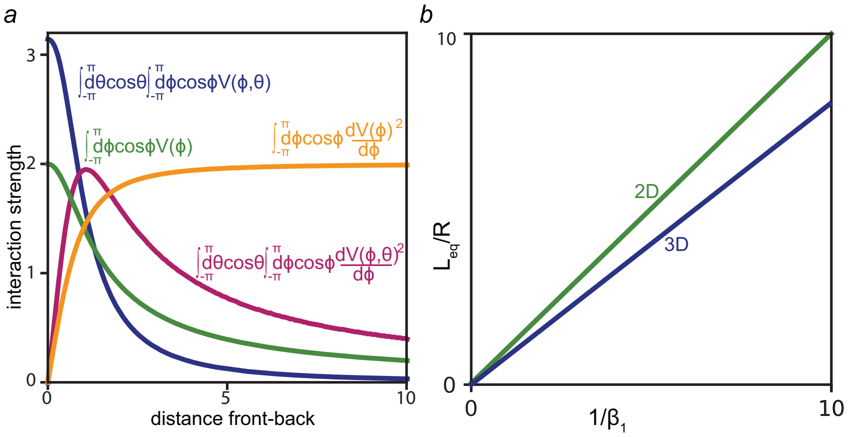

Fig. S3. **Interaction strength in 2D and 3D.** a. Influence of the parameters of the equation on an observer looking at an object at different distances front-back where the distance left-right and up-down are both equal to zero in 2D (green and orange) and in 3D (blue and purple). B. Equilibrium distance, L_{eq}/R as a function of the parameter $1/\beta_1$ for both 2D (in green) and 3D in blue.

between repulsion and attraction in 3D is still correlated linearly with $1/\beta_1$ but the resulting distance is closer than what we obtain in 2D.

A small set of numerical simulations in 3D has been realized for $N = 210, 20, 50, 100$. Each individual agents is a sphere of radius $R = 0.5$, self-propelled with a velocity $\mathbf{v} = \mathbf{e}_\psi$, and the speed relaxation rate is $\gamma = 0.1$. The time step between two updates of the velocity is $\Delta t = 0.1$ while the whole simulation runs until a time $T_{end} = 1000$. In all simulations $\alpha_1 = \beta_1 = 0.1$ so that the equilibrium distance in the front-back and left right direction are the same, accounting for an equilibrium distance of 5. Furthermore, we choose the following parameters are taken so that polarization is observed with N=50 individuals in absence of movements in the z direction, $\alpha_0 = 5$, $\beta_0 = 2$ and $\lambda_0 = 10$. For this set parameters, range of values of $\lambda_1$have been explored to study the influence of the equilibrium distance in the z direction spanning multiple order of magnitude,

$$\lambda_0 = 0.005, 0.001, 0.05, 0.075, 0.1, 0.15, 0.2, 0.5, 1.0, 2.0, 5.0$$

Each sets of parameters is then replicated 5 times so that a total of 275 simulations have been performed.

As in 2D. due to the formulation of the minimal model, ray-casting is not required for the calculation of individual visual projections. Instead, the visual projection field of each individual, V_i, is considered as a square surface with $\phi \in [-\pi, \pi]$, and $\theta \in [-\pi/2, \pi/2]$ constituted of 513×257 elements so that $\Delta\theta = \pi/257$. however, it is not possible to unwrap a sphere on a flat surface. $\Delta\phi$ needs to be corrected to account for this difference so that $\Delta\phi = \cos\theta\Delta\theta$. $V_i(\phi, \theta)$ is initialized at 0 at each time step. The visual field is then updated by considering the relative position of each individual j, in the referential of the focal individual i oriented in the direction of the movement ψ_i, (x_j^i, y_j^i, z_j^i). The position in the visual field is recovered by computing the relative angle of each individual $\phi_j^i = \arctan y_j^i/x_j^i$, with the extension $\Delta\phi_j^i = \arctan R/d_j^i$, where $d_i^j = \sqrt{{x_j^i}^2 + {y_j^i}^2}$. The values of the visual field are then updated so that $V_i(\phi, \theta) = 1$ in the range $(\phi_j^i - \Delta\phi_j^i, \phi_j^i + \Delta\phi_j^i)$. As the visual field is binary, there is no need to considered supplementary calculation to account for occlusion. The velocity and turning rate of each individual at time $t + \Delta t$ is then updated according to the discretized equations of motions:

$$v_i(t+\Delta t) = v_{\psi i}(t) + \Delta t \sum_{\theta=-\pi/2}^{\pi/2} \cos\theta\cos\theta\Delta\theta \sum_{\phi=-\pi}^{\pi} \cos\phi\alpha_0 \left(V\Delta\phi + \alpha_1\partial_\phi V\right) \quad \text{(S43)}$$

$$\psi_i(t+\Delta t) = \psi_i(t) + \Delta t \sum_{\theta=-\pi/2}^{\pi/2} \cos\theta\cos\theta\Delta\theta \sum_{\phi=-\pi}^{\pi} \sin\phi\beta_0 \left(V\Delta\phi + \beta_1\partial_\phi V\right) \quad \text{(S44)}$$

$$v_{zi}(t+\Delta t) = \psi_i(t) + \Delta t \sum_{\theta=-\pi/2}^{\pi/2} \cos\theta\sin\theta\Delta\theta \sum_{\phi=-\pi}^{\pi} \lambda_0 \left(V\Delta\phi + \beta_1\partial_\phi V\right) \quad \text{(S45)}$$

The maximal distance observed in the plane(x,y), $d_{\psi,max}$ is measured by taking the maximal distance of an individual measured from the center of mass of the group at each time step and updating for all the time of the simulation

$$d_{\psi,max} = \min_{t=0}^{t=t_{end}} \left(\max_{i=1,j=1,i\neq j}^{i=N,j=N} \sqrt{(x_i^j(t)^2 + y_i^j(t)^2)+} \right) \quad \text{(S46)}$$

The mean polarization, p is measured as the average polarization between all individuals at each time step in the plane (x, y), averaged on the last 10% of each simulation, so in the temporal range $t = (900, 1000)$,

$$p = \frac{1}{100} \sum_{t=900}^{1000} \left(\frac{1}{\sum_{k=1}^{k=N-1} k} \sum_{i=1}^{i=N} \sum_{j=i,j\neq i}^{j=N} \frac{\mathbf{v_i}(t)\mathbf{v_j}(t)}{v_i(t)v_j(t)} \right) \tag{S47}$$

The average maximal distance observed in the plane (x, y), $d_{\psi,max}$ is measured as the average 10% of each simulation, so in the temporal range $t = (900, 1000)$, of the maximal distance of an individual measured from the center of mass of the group at each time step and updating for all the time of the simulation

$$d_{\psi max} = \frac{1}{100} \sum_{t=900}^{1000} \left(\min_{i=1,j=1,i\neq j}^{i=N,j=N} \sqrt{(x_i^j(t)^2 + y_i^j(t)^2)} \right) \tag{S48}$$

The average maximal distance observed in the direction z, d_{zmax} is measured as the average 10% of each simulation, so in the temporal range $t = (900, 1000)$, of the maximal distance of an individual measured from the center of mass of the group at each time step and updating for all the time of the simulation

$$d_{zmax} = \frac{1}{100} \sum_{t=900}^{1000} \left(\min_{i=1,j=1,i\neq j}^{i=N,j=N} \sqrt{(z_i^j(t)^2)} \right) \tag{S49}$$

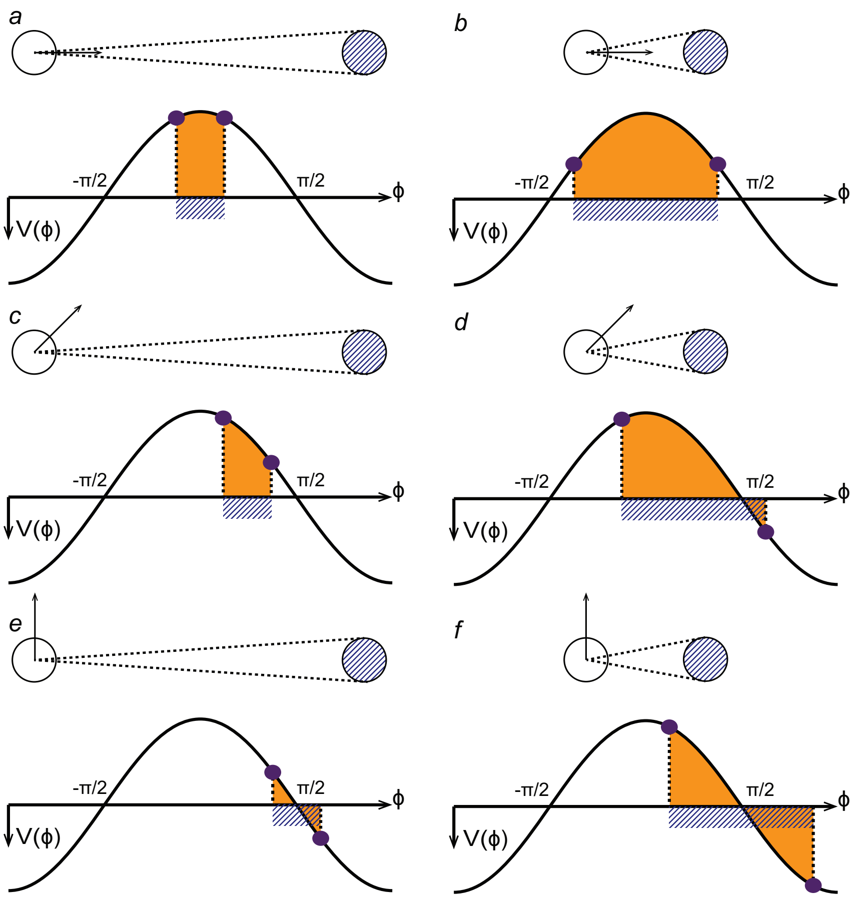

Fig. S4. Effects of the terms of the Eq. 3 (main text) on a focal observer according to the relative position the other disk (blue). This is a complement to figure 2. Here The white disk is looking at the blue disk with an eye positioned in the center, but with different relative angles (0, $\pi/4$ and $\pi/2$) between the direction of the observer and the position of the blue disk in the referential of the white disk. When the object is in front, a and b, this situation has been described in Figure 2. If the observer rotates around its center, the object moves in his visual field (c,d,e, and f). However, if the distance is kept constant, the subtended angle remains the same. When the object is directly perpendicular to the observer, both the contribution of the subtended angle and the edges vanish. Indeed, the cosine is anti-symmetrical around $\pi/2$, so the integral subtract two surfaces of the same area, while the edges are summed over two points of opposite values. In intermediary cases ($\pi/4$, b and c), the surface given by the integral of cosine over the subtended angle is reduced. Furthermore, if the object is close enough, part of the integration might be negative reducing even further the contribution of the subtended angle. A similar analysis can be made for the edges of the object.
